# Transcriptome analysis indicates TFEB1 and YEATS4 as regulatory transcription factors for drug resistance of ovarian cancer

**DOI:** 10.18632/oncotarget.5208

**Published:** 2015-08-17

**Authors:** Yi Rang Kim, Mi Sung Park, Ki Hwan Eum, Juhee Kim, Jeong Won Lee, Taejeong Bae, Dae Ho Lee, Jin Woo Choi

**Affiliations:** ^1^ Department of Hemato-Oncology, Yuseong Sun Hospital, Daejeon, Republic of Korea; ^2^ Institute for Metabolic Disease, School of Medicine, Wonkwang University, Iksan, Jeonbuk, Republic of Korea; ^3^ Wonkwang Institute of Interfused Biomedical Science and Dental Research Institute, School of Dentistry, Wonkwang University, Iksan, Chonbuk, Republic of Korea; ^4^ Department of Obstetrics and Gynecology, Samsung Medical Center and Samsung Biomedical Research Institute, Sungkyunkwan University School of Medicine, Seoul, Republic of Korea; ^5^ Division of Biomedical Statistics and Informatics, Department of Health Sciences Research, Mayo Clinic, Rochester, MN, USA; ^6^ Department of Internal Medicine, Wonkwang University School of Medicine and Hospital, Iksan, Jeonbuk, Republic of Korea; ^7^ Advanced Institute of Convergence Technology, Seoul National University Suwon Gyeonggi-do, Korea

**Keywords:** cancer, transcription factor, drug resistance, bioinformatics

## Abstract

Ovarian cancer is an intractable disease because patients with ovarian cancer frequently develop drug resistance after long-term chemotherapy. Despite the availability of cumulative information on drug-resistant patients, strategies to reverse drug resistance have still not been established. In this study, we analyzed drug resistance-associated transcription factors (TFs) in ovarian cancer. Gene expression profiles of 15 drug-resistant and 11 drug-sensitive patients with ovarian cancer were compared. Our results showed that TFs TFEB1 and YEATS4 regulated the expression of downstream target genes. These 2 TFs have already been implicated in tumorigenesis or metastasis. To our knowledge, this is the first study to evaluate the involvement of these TFs in drug resistance of ovarian cancer. Interestingly, 70% knockdown of each of these TFs with siRNAs resulted in approximately 20%∼30% recovery of drug sensitivity. Further, combination treatment of ovarian cancer cells with *TFEB1* and *YEATS4* siRNAs resulted in 35% reversal of drug resistance. The effect of these TFs on chemoresistance seemed to be associated with intrinsic apoptosis-related pathways, such as p53 activation, and not with the suppression of drug transport. Thus, we suggest a novel approach to reverse chemoresistance of ovarian cancer by suppressing TFEB1 and YEATS4.

## INTRODUCTION

Ovarian cancer is one of the most life-threatening cancers in women worldwide because this rapidly proliferating cancer compresses visceral organs and is only temporarily chemosensitive, with a cure rate of only 30% [1]. After chemotherapy, drug-resistant ovarian cancer recurs in approximately 25% patients within 6 months, with an overall 5-year survival rate of 30% [2]. Treatment of epithelial ovarian cancer involves a combination of surgery and chemotherapy with taxanes and platinum [3]. However, most patients eventually develop severe drug resistance after long-term treatment.

Drug resistance develops through a few common mechanisms. For instance, overexpression of *MDR1*, which encodes P-glycoprotein, hampers the retention of taxanes and other cationic drugs [4, 5]. Further, cytoplasmic aldehyde dehydrogenase directly binds to anticancer drugs and deactivates them. Alternatively, drug resistance can also develop after treatment with specific drugs. Platinum drugs attach to DNA duplexes in the nucleus of cancer cells and inhibit DNA replication [3, 6]. Platinum resistance is mainly associated with the alteration of intrinsic apoptosis pathways. Taxanes, such as, paclitaxel are standard cytotoxic drugs for treating ovarian cancer. Taxanes target microtubules and block mitosis and cell proliferation. Taxane resistance occurs because of genetic or post-translational modifications in microtubule formation and cell cycle [4]. A study by Sherman-Baust *et al*. showed that 845 genes were altered in at least one drug-resistant phenotype of ovarian cancer [7]. Further, 460, 366, and 337 genes were aberrantly altered in ovarian cancer cells resistant to cisplatin, doxorubicin, and paclitaxel, respectively [7]. However, only 62 of these genes were common among cells resistant to all the 3 drugs. Because targeting every gene listed above is practically impossible, it may be efficient to target genes encoding the most upstream transcription factors (TFs;[7, 8]. Regulation of TFs may provide an economical method to change cellular physiology with minimum effort [9]. TFs bind to specific DNA sequences and control their transcription rate [10, 11]. A single TF manages various downstream target genes. Therefore, modulation of a single TF may affect the expression of several downstream genes, indicating that TFs are promising druggable targets [9]. Understanding the complex network underlying chemoresistance and determining upstream TFs may provide strategies to overcome or prevent drug resistance.

This study used algorithm for the reconstruction of accurate cellular networks (ARACNE) to establish a network of TFs by using microarray data from tissues of chemoresistant patients [8]. ARACNE, a newly developed transcriptome analysis program, analyzes functional associations between metabolic networks. ARACNE analyzes the expressions of all genes in patients with a particular disease and identifies genes associated with the upregulation or downregulation of specific gene sets, thus predicting functional relationships among these genes [8].

Transcriptional network isolated from the tissues of drug-resistant patients suggested that TFEB1 and YEATS4 could be targeted to ameliorate drug resistance. Although these TFs are implicated in tumorigenesis or metastasis, their association with drug resistance has not been investigated thus far. In this study, by using ARACNE we found that TFEB1 and YEATS4 were the most upstream TFs that regulated genes implicated in drug resistance [8, 10, 11]. This result may be used for developing combination drug therapies or novel drugs to prevent drug resistance and to treat drug-resistant patients with ovarian cancer who use long-term chemotherapy.

## RESULTS

### Prediction of transcriptional network and TFs involved in the development of chemoresistance

**Figure 1:**
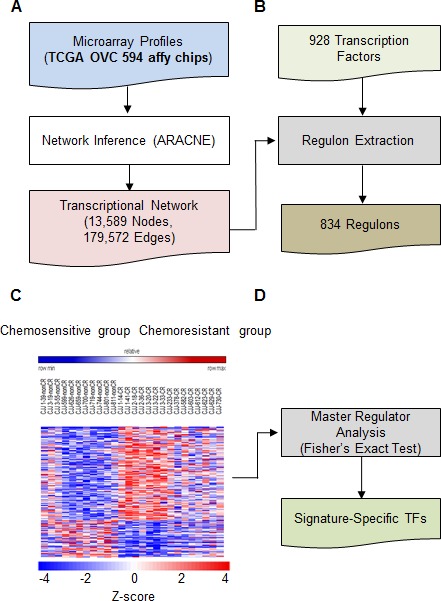
Overview of transcriptional network involved in the development of drug resistance **A.,** ARACNE was used to identify the transcriptional network in approximately 594 ovarian cancer samples obtained from TCGA. **B.** Regulon extraction using previously defined TFs. **C.** Expression signatures of genes involved in drug resistance of ovarian cancer cells were obtained using Affymetrix GeneChip Human Gene 1.0 ST oligonucleotide arrays. **D.** Genes overlapping between regulons in B and gene signatures in C were enriched and statistically analyzed.

To understand chemoresistance-related transcriptional network in ovarian cancer, gene expression signatures of tissue samples from 594 patients with ovarian cancer were obtained from TCGA, and the transcriptional network was determined using ARACNE (Figure 1A and Data S1). The previously listed 928 TFs were enriched in ovarian cancer cells (Figure 1B) [8]. These gene expression signatures were used to obtain drug resistance signatures of patients with ovarian cancer. Original data were rearranged based on tumor recurrence after chemotherapy [12]. Briefly, to determine the expression of different genes in drug-resistant ovarian cancer cells, cDNA was generated from RNA obtained from 11 drug-sensitive and 15 drug-resistant patients with ovarian cancer (Figure 1C and Data S2) [12]. Differentially expressed genes (DEGs) were determined based on the difference of > 1.5 folds and significance of > 95% (Figure 1D, middle panel).

**Figure 2:**
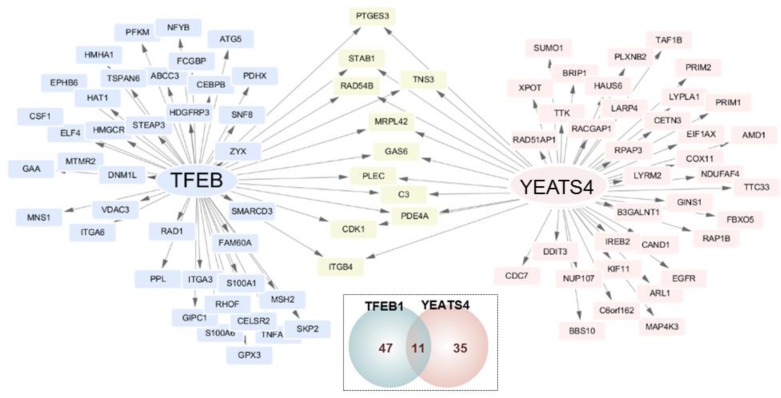
Transcriptional network of genes involved in drug resistance in ovarian cancer TFEB1 and YEATS4 were identified as the master regulators. Their target genes are shown along with the genes altered in ovarian cancer cells. The TFs are indicated in the middle of nodes. Grey arrow indicates several target genes. Blue nodes represent target genes of TFEB1, pink nodes represent target genes of YEATS4, and yellow nodes represent target genes linked with both the TFs. Mode of regulation was determined using Spearman's rank correlation between expression of the TF and its target gene. The small Venn diagram shows the total number of genes regulated by each TF.

Two TFs YEATS4 and TFEB1 were anticipated to be the common upstream regulators that conferred drug resistance to ovarian cancer cells. Effect of these TFs on downstream genes was assessed. It was found that TFEB1 was correlated with the expression of 47 downstream genes and YEATS4 was linked to the expression of 35 downstream genes. Further, 11 genes were regulated by both YEATS4 and TFEB1 (Figure 2).

### *In vitro* validation of TFEB1 on reversal effect of drug resistance

**Figure 3:**
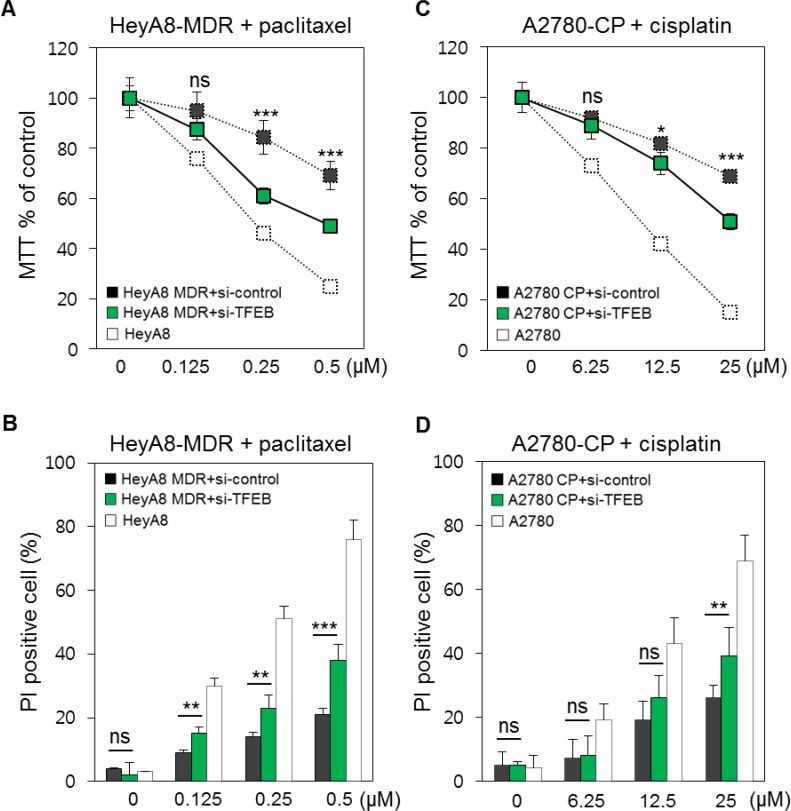
*In vitro* validation of the involvement of TFEB1 in drug resistance **A.** HeyA8-MDR cells were treated with *TFEB1* siRNA. Apoptosis was monitored using increasing doses of paclitaxel. Drug-sensitive HeyA8 cells and control siRNA-treated HeyA8-MDR cells were used as controls. **B.** Proportion of apoptotic cells was confirmed using PI staining by comparing with the control cells. **C.** A2780-CP cells were treated with *TFEB1* siRNA. Drug-sensitive A2780 cells and control siRNA-treated A2780-CP cells were used as controls. **D.** Proportion of apoptotic cells after paclitaxel treatment was validated using PI staining as described above. ns, not significant; *P < 0.05; **P < 0.01; ***P < 0.001.

To determine whether suppression of TFEB1 reversed chemoresistance of ovarian cancer cells, we prepared HeyA8-MDR, a paclitaxel-resistant cell line derived from HeyA8, and A2780-CP, a cisplatin-resistant cell line derived from A2780. We treated HeyA8-MDR and A2780-CP cells with *TFEB1* siRNA (Figure S1), followed by treatment with paclitaxel and cisplatin, respectively. Apoptosis was monitored using the MTT assay. siRNA-mediated suppression of *TFEB1* in HeyA8-MDR cells recovered paclitaxel (0.25 and 0.5 μM) sensitivity by approximately 20% compared with that in control siRNA-treated HeyA8-MDR cells (Figure 3A). Apoptosis was confirmed using PI staining. *TFEB1* siRNA-treated HeyA8-MDR cells showed increased apoptosis after paclitaxel treatment compared with control siRNA-treated HeyA8-MDR cells. However, a difference was observed between drug-sensitive HeyA8 cells and *TFEB1* siRNA-treated HeyA8-MDR cells (Figure 3A and 3B). Reduced proliferation of *TFEB1* siRNA-treated A2780-CP cells was measured using MTT assay (Figure 3C). The results obtained were validated using PI staining. Higher dose of paclitaxel significantly increased the apoptosis of *TFEB1* siRNA-treated A2780-CP cells (Figure 3D). Increase in the apoptosis of *TFEB1* siRNA-treated A2780-CP cells was lower than that of *TFEB1* siRNA-treated HeyA8-MDR cells.

### *In vitro* validation of YEATS4 on reversal effect of drug resistance

**Figure 4:**
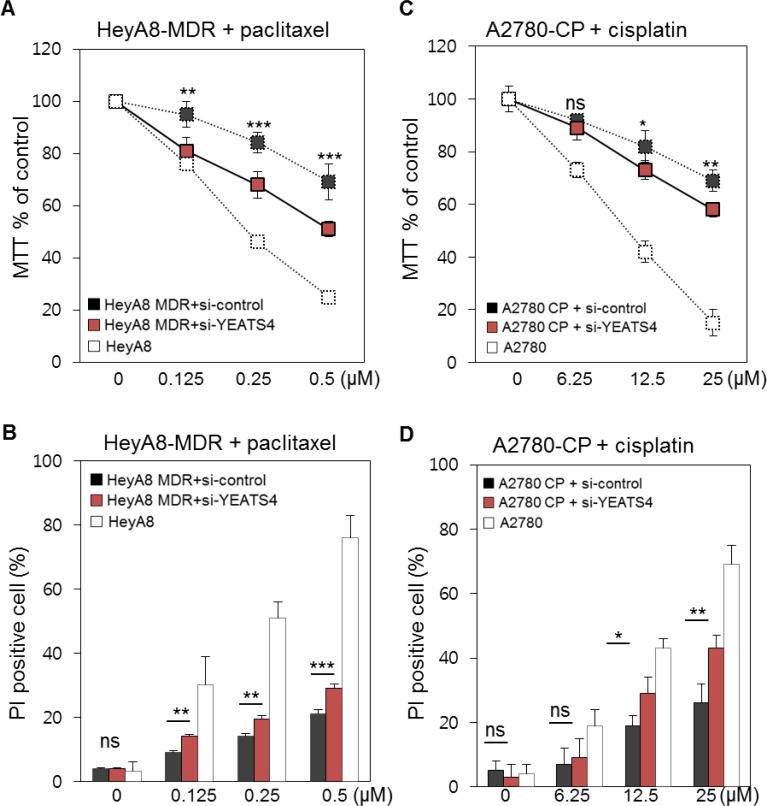
*In vitro* validation of the involvement of YEATS4 in drug resistance **A.** HeyA8-MDR cells were treated with *YEATS4* siRNA. Apoptosis was monitored using increasing doses of paclitaxel. Drug-sensitive HeyA8 cells and control siRNA-treated HeyA8-MDR cells were used as controls. The control value used in Figure 3 was repeated as they are originally performed at the same time. **B.** Proportion of apoptotic cells was confirmed using PI staining by comparing with the control cells. **C.** A2780-CP cells were treated with *YEATS4* siRNA. Drug-sensitive A2780 cells and control siRNA-treated A2780-CP cells were used as controls. **D.** Proportion of apoptotic cells after paclitaxel treatment was confirmed using PI staining as described above. ns, not significant; *P < 0.05; **P < 0.01; ***P < 0.001.

Next, we determined whether YEATS4 decreased drug resistance of ovarian cancer cells by treating HeyA8-MDR and A2780-CP cells with *YEATS4* siRNA. Efficacy of siRNA treatment was checked (Figure S2). Treatment with *YEATS4* siRNA increased the apoptosis of HeyA8-MDR cells, with approximately 20% difference in apoptotic cells between *YEATS4* siRNA- and control siRNA-treated cells (Figure 4A). Treatment with 0.5 μM paclitaxel decreased MTT percentage (MTT%) of *YEATS4* siRNA-treated HeyA8-MDR cells by 50%. This result was validated using PI staining, with *YEATS4* siRNA-treated HeyA8-MDR cells showing increased apoptosis compared with control cells (Figure 4B). *YEATS4* siRNA-treated A2780-CP cells also responded to cisplatin, and the responsiveness increased with an increase in the dose of cisplatin from 6.25 to 25 μM (Figure 4C). These results were validated using PI staining. Cisplatin concentration of > 12.5 μM significantly increased the apoptosis of *YEATS4* siRNA-treated A2780-CP cells compared with that of control siRNA-treated A2780-CP cells (Figure 4D).

HeyA8-MDR and A2780-CP cells were treated with a combination of *TFEB1* and *YEATS4* siRNAs. Although the apoptotic rate of cells treated with the combination of siRNAs was higher than that of cells treated with control siRNAs, the increase in the level of apoptosis compared with that of cells treated with either *TFEB1* or *YEATS4* siRNA was unclear (Figure S3A and B). Apoptosis of cells treated with both *TFEB1* and *YEATS4* siRNAs was compared with that of cells treated with either *TFEB1* or *YEATS4* siRNA. Although the proportion of PI-stained cells, which indicated dead cells, was slightly higher among cells treated with both *TFEB1* and *YEATS4* siRNAs than among cells treated with either *TFEB1* or *YEATS4* siRNA, the difference was < 5% and even it is not statistically significant partially in HeyA8-MDR. *TFEB1* and *YEATS4* siRNA-treated A2780-CP cells showed slightly higher apoptosis rate than *TFEB1* and *YEATS4* siRNA-treated HeyA8-MDR cells (Figure S3C and D).

### Alteration of gene expression by TFEB1 and YEATS4

**Figure 5:**
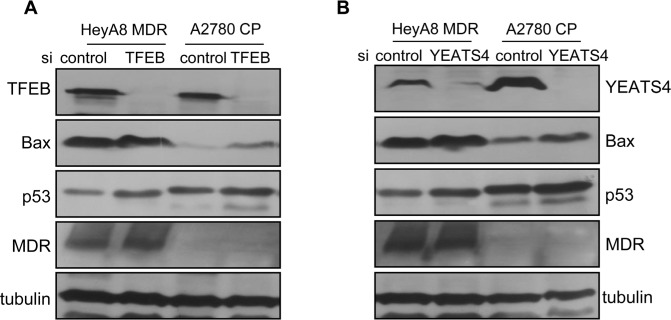
Molecular changes after suppression of TFEB1 or YEATS4 **A.** Western blotting was used to compared the expression of genes involved in drug resistance between *TFEB1* siRNA- and control siRNA-treated HeyA8-MDR and A2780-CP cells. TFEB1 level was measured to determine the efficacy of siRNA treatment. **B.** Western blotting was used to compared the expression of genes involved in drug resistance between *YEATS4* siRNA- and control siRNA-treated HeyA8-MDR and A2780-CP cells. Tubulin was used as a loading control.

Further, western blotting was performed to determine the altered gene expression profiles of *TFEB1*- and *YEATS4*-knockdown cells. Suppression of TFEB1 increased the levels of p53 and Bax, the intrinsic apoptosis-related proteins, in *TFEB1* siRNA-treated HeyA8-MDR and A2780-CP cells compared with those in control cells (Figure 5A and 5B). Baseline levels of p53 and Bax were slightly higher in A2780-CP cells than in HeyA8-MDR cells. Expression of P-glycoprotein, which is associated with drug influx, was relatively stable in HeyA8-MDR cells but was not detected in A2780-CP cells.

## DISCUSSION

Studies on the early detection of ovarian cancer and determination of new therapeutic approaches to reduce mortality have been largely unsuccessful because the origin and pathogenesis of epithelial ovarian cancer are poorly understood [13]. Ovarian cancer can be cured as long as cancer cells do not acquire drug resistance. However, ovarian cancer is one of the most incurable cancers because ovarian cancer cells frequently acquire drug resistance. Delaying the occurrence of drug resistance or restoring drug sensitivity may allow effective treatment of this cancer. In this study, we analyzed the transcriptional network of ovarian cancer by using a previously published algorithm. This algorithm predicted TFEB1 and YEATS4 as the most upstream TFs that conferred drug resistance. To determine the genes altered in ovarian cancer cells, we obtained gene expression profiles of 594 patients with ovarian cancer from TCGA. Next, we enriched these genes with other DEGs that were determined using tissues obtained from drug-sensitive and drug-resistant patients with ovarian cancer. Thus, there might be a bias in the initially utilized gene pool. Although we may have missed other TFs, TFEB1 and YEATS4 are not likely to be mispredicted as being involved in conferring drug resistance to ovarian cancer cells.

TFEB1 significantly increases the transcriptional activation of many lysosome- and autophagy-related genes. Because autophagy partially results in drug resistance [14], it can be logically inferred that suppressing TFEB1 expression can compensate effect drug resistance. However, isolation of TFEB1 as a regulator of drug resistance has not been reported thus far. Especially, limited studies have been performed on TFEB1 for restoring drug sensitivity. TFEB1 triggers the overexpression of lysosome-related genes by directly binding to their promoters [15]. This significantly increases the biogenesis of lysosomes [16]. TFEB1 plays an important role in regulating autophagy. Autophagy allows cells to degrade their own components and to reuse vital molecules during malnutrition. Deficiency of endogenous TFEB1 reduces the number of autophagosome [17]. Thus, autophagy confers drug resistance to cancer cells by suppressing the action of drugs entering these cells. Thus, suppression of TFEB1 may decrease the number of autophagosomes. Further, TFEB1 supports cell homeostasis. TFEB1 shuttles between the cytoplasm and lysosomes in well-nourished cells; however, it is transported from the cytoplasm into the nucleus in malnourished cells. Thus, TFEB1 controls the number of lysosomes in the cytoplasm based on the nutrition status of cells [15, 16].

YEATS4 is essential for cell growth and viability [18]. YEATS4 has an N-terminal TFIIF domain that is conserved in all YEATS family proteins [19]. This family includes proteins such as yeast Yaf9, TAF14, and SAS5 as well as proteins implicated in human cancers [20]. Although YEATS4 is known to suppress p53 activity in the nucleus [10] and is upregulated in non-small-cell lung cancer, its involvement in the development of drug resistance has not been reported to date [20]. Human YEATS4 is an imperfect TF because it does not contain DNA-binding sites. Moreover, it is associated with DNA translocation and shows high relativity with yeast AF-9 and ENL proteins. YEATS4 associates with NuMA, KIAA1009, and PFDN1 [18]. Combination treatment with *TFEB1* and *YEATS4* siRNAs did not increase the apoptosis of HeyA8-MDR and A2780-CP cells compared with treatment with individual siRNAs (Figure S3), indicating that TFEB1 partially overlapped YEATS4 while inducing drug resistance. Because YEATS4 does not have DNA-binding sites, its transcriptional contribution to the development of drug resistance may depend on TFEB1. Further, of the 93 ovarian cancer genes regulated by TFEB1 and YEATS4, 11 (12%) were commonly associated by both these TFs (Figure 2). Thus, suppression of both TFEB1 and YEATS4 by combination treatment with the respective siRNAs did not provide clear results.

Although knockdown of *TFEB1* and *YEATS4* increased the expression of p53 compared with its baseline level, expression of *MDR* was unchanged (Figure 5). This result suggested that TFEB1 and YEATS4 were only slightly involved in the transport of drugs into cells and were largely involved in potentiating intrinsic apoptosis pathways upon drug treatment.

Taken together, these results suggested that TFEB1 and YEATS4 were the key TFs that developed drug resistance. These results may be used to increase the effectiveness of drugs used for treating ovarian cancer.

## METHODS AND MATERIALS

### Analysis of transcriptional network

Transcriptional network of OVC was constructed using OVC gene expression data (HT_HG-U133A, level 1) of 594 patients that was obtained from The Cancer Genome Atlas (TGCA). Raw CEL files were downloaded from the TCGA Data Portal (http://tcga-data.nci.nih.gov/tcga) and were normalized using MAS5 algorithm (affy package in R/Bioconductor). Next, ARACNE (http://wiki.c2b2.columbia.edu/califanolab/index.php/Software/ARACNE) was used to determine interactions between previously known TFs and other genes in the TCGA OVC data. We used 928 TF genes that were previously used in a study by Carro *et al* [8]. In all, 100 bootstrapped networks were generated and were merged into a consensus network by using a consensus voting method based on statistically significant number of inferred interactions in bootstrapping steps. This probe-level consensus network was merged with a gene-level network. The final gene-level OVC transcriptional network included 13,589 genes (834 TFs) and 179,572 interactions.

### Analysis of master regulators

A list of 928 human TF genes used by Carro *et al* was obtained [8]. These 928 TFs were mapped against 2155 probe sets of Affymetrix HG-U133 Plus 2.0 GeneChip array for obtaining regulon structure from the transcriptional network for further analysis. Each target gene of 834 TF regulons was compared with OVC chemoresistance signature to identify upstream TFs with specific signatures. Fisher's exact test for overlaps between target and signature genes was performed, and TFs showing statistically significant results (FDR < 0.05) were selected. These TFs were ranked according to the number of target genes that were linked with signature genes in each regulon.

### Cell lines and materials

Epithelial ovarian cancer cell lines HeyA8, HeyA8-MDR, A2780, and A2789-CP were provided by Dr. Anil K. Sood of the MD Anderson Cancer Center. These cell lines were cultured in RPMI-1649 (Hyclone, USA) supplemented with 10% FBS and gentamicin (250 ng/mL). HeyA8-MDR cells were cultured in RPMI-1649 supplemented with 300 ng/mL paclitaxel to retain their chemoresistance. Paclitaxel, alsterpaullone, propidium iodide (PI), and Hoechst 33342 were purchased from Sigma-Aldrich. Cisplatin was obtained from LC Laboratories (MA, US), and siRNAs were purchased from Bioneer Inc. (South Korea). Sequences *TFEB1* and *YEATS4* siRNAs were 5′-GUACCUGUCCGAGACCUAU-3′ and 5′-CUCAUGAGAACUUGGUAGU-3′, respectively. SiRNAs were used at a concentration of 10 μM.

### Cell death assay

Cell growth was measured using 3-(4,5-dimethylthiazol-2-yl)-2,5-diphenyltetrazolium bromide (MTT) assay (Promega, Ltd.), according to the manufacturer's protocol. Briefly, cells were seeded in 96-well plates at a density of siRNA-treated 5 × 10^3^ cells per well. After treatment with cisplatin or paclitaxel, the cells were incubated with 5 mg/mL MTT for 4 h. Next, the medium was removed, and the cells were incubated with 150 μL solubilization solution and stop solution at 37°C for 4 h. Absorbance of the reaction solution was measured at 570 nm. Rate of cell growth inhibition was calculated using the following formula: (1 - absorbance of experimental group/absorbance of control group) × 100.

Apoptosis was confirmed by double staining the cells with 10 μM Hoechst 33342 and 1 μM PI for 20 min at 37°C. After washing twice with serum-free medium, the cells were photographed under a fluorescence microscope (Nikon, Japan). PI-stained cells were counted using a cell counter under the fluorescence microscope at 100× magnification. Total number of PI-stained cells in 3 representative fields of view per well were quantified for each treatment group to estimate the percentage of PI-stained cells out of the total number of Hoechst 33342-stained cells.

### Western blotting

Cells were lysed at 4°C in RIPA buffer (20 mM Tris-HCl [pH 7.5], 150 mM NaCl, 1 mM Na2EDTA, 1 mM EGTA, 1% Triton, 2.5 mM sodium pyrophosphate, 1 mM b-glycerophosphate, 1 mM sodium orthovanadate, 1 μg/mL leupeptin, and 1 mM PMSF [Cell Signaling Technology, Inc.]) Cell lysates were electrophoresed on SDS-PAGE gel to separate proteins. The separated proteins were transferred onto a PVDF membrane and were assessed using antibodies against MDR (C219; Novus Biologicals), Bax (E63; Abcam), p53 (DO-1; Santa Cruz Biotechnology), and β-actin (C4; Santa Cruz Biotechnology).

## SUPPLEMENTARY MATERIAL FIGURES AND TABLES






