# Supplementary material for: Transcriptome analysis indicates TFEB1 and YEATS4 as regulatory transcription factors for drug resistance of ovarian cancer
Source: Oncotarget. 2015 Aug 17;6(31):31030–8. doi: 10.18632/oncotarget.5208 (PMC4741586; doi:10.18632/oncotarget.5208)
Supplement: Supplementary file 1 [file oncotarget-06-31030-s001.pdf]

Transcriptome analysis indicates TFEB1 and YEATS4 as regulatory transcription factors for drug resistance of ovarian cancer

Supplementary Material

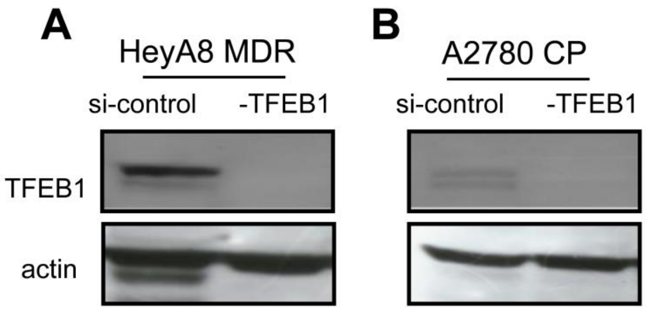

Supplementary Figure 1. Efficacy of *TFEB1* siRNA. HeyA8-MDR and A2780-CP cells were treated with 10 μM *TFEB1* siRNA. Cells were harvested at 48 h after siRNA treatment, and cell lysates were assessed using western blotting. Expression levels of tubulin (Santa Cruz Biotechnology) and TFEB1 (Santa Cruz Biotechnology) were measured.

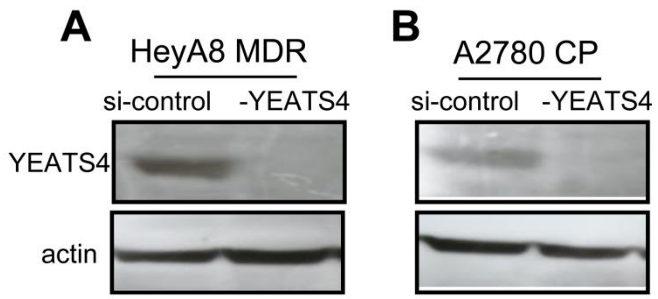

Supplementary Figure 2. Efficacy of *YEATS4* siRNA. HeyA8-MDR and A2780-CP cells were treated with 10 μM *YEATS4* siRNA. Cells were harvested at 48 h after siRNA treatment, and cell lysates were assessed using western blotting. Expression levels of tubulin (Santa Cruz Biotechnology) and YEATS4 (Santa Cruz Biotechnology) were measured.

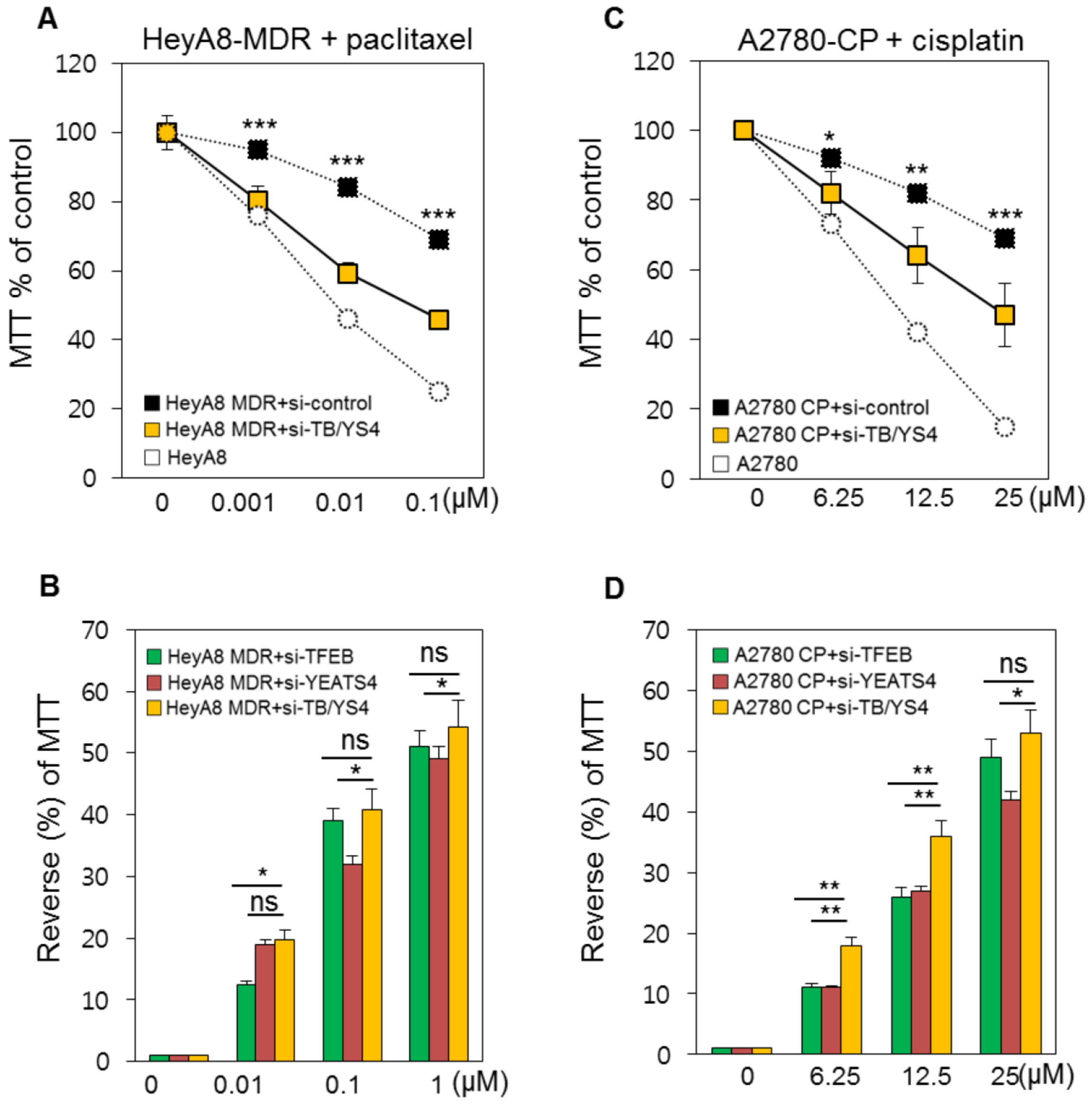

Supplementary Figure 3. Effect of combination treatment with both *TFEB1* and *YEATS4* siRNAs. A, HeyA8-MDR cells were treated with *TFEB1* and *YEATS4* siRNAs, and induction of apoptosis by using increasing doses of paclitaxel was measured. B, proportion of apoptotic cells was confirmed using PI staining. Apoptosis due to treatment with either *TFEB1* or *YEATS4* siRNA was used as control. C, A2780-CP cells were treated with *TFEB1* and *YEATS4* siRNAs, and induction of apoptosis by using increasing doses of paclitaxel was measured. D, proportion of apoptotic cells was confirmed using PI staining. ns, not significant; **P* < 0.05; ***P* < 0.01; ****P* < 0.001.
