# Supplementary material for: Transcriptome analysis indicates TFEB1 and YEATS4 as regulatory transcription factors for drug resistance of ovarian cancer
Source: Oncotarget. 2015 Aug 17;6(31):31030–8. doi: 10.18632/oncotarget.5208 (PMC4741586; doi:10.18632/oncotarget.5208)
Supplement: Supplementary file 2 [file oncotarget-06-31030-s002.pdf]

Gene Accession	Gene Symbol	Gene Description	log2 ratio
NM_016247	IMPG2	interphotoreceptor matrix proteoglycan 2	1.76459424
NM_002909	REG1A	regenerating islet-derived 1 alpha	1.48714053
NM_001056	SULT1C2	sulfotransferase family, cytosolic, 1C, member 2	1.46609364
NM_006183	NTS	neurotensin	1.45018641
NM_004378	CRABP1	cellular retinoic acid binding protein 1	1.37032278
NM_012152	LPAR3	lysophosphatidic acid receptor 3	1.31553516
NM_014585	SLC40A1	solute carrier family 40 (iron-regulated transporter), member 1	1.30543571
NM_031422	CHST9	carbohydrate (N-acetylgalactosamine 4-0) sulfotransferase 9	1.24744614
NM_020775	KIAA1324	KIAA1324	1.19516519
NM_024626	VTCN1	V-set domain containing T cell activation inhibitor 1	1.19146936
NM_032918	RERG	RAS-like, estrogen-regulated, growth inhibitor	1.17562426
NM_004615	TSPAN7	tetraspanin 7	1.16990442
NM_000921	PDE3A	phosphodiesterase 3A, cGMP-inhibited	1.1624665
NM_001584	MPPED2	metallophosphoesterase domain containing 2	1.15424384
NM_001657	AREG	amphiregulin	1.14426685
NM_002310	LIFR	leukemia inhibitory factor receptor alpha	1.14385228
NM_006614	CHL1	cell adhesion molecule with homology to L1CAM (close homolog of L1)	1.11501777
NM_138788	TMEM45B	transmembrane protein 45B	1.08133516
NM_001786	CDC2	cell division cycle 2, G1 to S and G2 to M	1.06138146
NM_002644	PIGR	polymeric immunoglobulin receptor	1.01224776
NM_024636	STEAP4	STEAP family member 4	1.00814747
NM_014373	GPR160	G protein-coupled receptor 160	1.00787904
NM_004529	MLLT3	myeloid/lymphoid or mixed-lineage leukemia (trithorax homolog, Drosoph	1.00673925
NM_032303	HSDL2	hydroxysteroid dehydrogenase like 2	0.99978757
NM_000898	MAOB	monoamine oxidase B	0.99363289
NM_004900	APOBEC3B	apolipoprotein B mRNA editing enzyme, catalytic polypeptide-like 3B	0.98504565
NM_002591	PCK1	phosphoenolpyruvate carboxykinase 1 (soluble)	0.98209981
NM_173081	ARMC3	armadillo repeat containing 3	0.98053071
NM_002988	CCL18	chemokine (C-C motif) ligand 18 (pulmonary and activation-regulated)	0.97559848
NM_002354	EPCAM	epithelial cell adhesion molecule	0.97249952
NM_021804	ACE2	angiotensin I converting enzyme (peptidyl-dipeptidase A) 2	0.94916822
NM_017912	HERC6	hect domain and RLD 6	0.9445463
NM_001123364	C6orf186	chromosome 6 open reading frame 186	0.92572587
NM_001100624	CENPN	centromere protein N	0.92568961
NM_024580	EFTUD1	elongation factor Tu GTP binding domain containing 1	0.92395541
NM_005063	SCD	stearoyl-CoA desaturase (delta-9-desaturase)	0.91695267
NM_002038	IFI6	interferon, alpha-inducible protein 6	0.9161406
NM_001874	CPM	carboxypeptidase M	0.91300218
NR_002754	RNU5E	RNA, U5E small nuclear	0.91053744
NM_001122659	EDNRB	endothelin receptor type B	0.90757316
NM_012253	TKTL1	transketolase-like 1	0.90724617
NM_015225	PRUNE2	prune homolog 2 (Drosophila)	0.90516309
NM_001038628	B3GALNT1	beta-1,3-N-acetylgalactosaminyltransferase 1 (globoside blood group)	0.90484702
NM_001657	AREG	amphiregulin	0.90152499
NM_003106	SOX2	SRY (sex determining region Y)-box 2	0.89768281

NM_005950	MT1G	metallothionein 1G	0.88975557
NM_015028	TNIK	TRAF2 and NCK interacting kinase	0.88927635
NM_004753	DHRS3	dehydrogenase/reductase (SDR family) member 3	0.88772279
NM_006547	IGF2BP3	insulin-like growth factor 2 mRNA binding protein 3	0.88365281
NM_001148	ANK2	ankyrin 2, neuronal	0.88183633
BC022252	C10orf57	chromosome 10 open reading frame 57	0.87561244
NR_024617	PART1	prostate androgen-regulated transcript 1	0.87481909
NM_001083619	GRIA2	glutamate receptor, ionotropic, AMPA 2	0.87402773
ENST00000299997	MGC9913	hypothetical protein MGC9913	0.87217567
NM_018492	PBK	PDZ binding kinase	0.86177965
NM_001130862	RAD51AP1	RAD51 associated protein 1	0.86126269
NR_023361	AP3S2	adaptor-related protein complex 3, sigma 2 subunit	0.86008562
NM_025130	HKDC1	hexokinase domain containing 1	0.85951359
NM_020640	DCUN1D1	DCN1, defective in cullin neddylation 1, domain containing 1 (S. cerevisiae)	0.85893533
NM_001001548	CD36	CD36 molecule (thrombospondin receptor)	0.85211478
NM_006061	CRISP3	cysteine-rich secretory protein 3	0.85134412
NM_198477	CXCL17	chemokine (C-X-C motif) ligand 17	0.8500587
NM_153234	LIX1	Lix1 homolog (chicken)	0.84840001
NM_025153	ATP10B	ATPase, class V, type 10B	0.84646683
NM_003503	CDC7	cell division cycle 7 homolog (S. cerevisiae)	0.84634386
NM_001001995	GPM6B	glycoprotein M6B	0.84571522
NM_001135091	MUC15	mucin 15, cell surface associated	0.84511965
NM_144765	MPZL2	myelin protein zero-like 2	0.84434057
NM_004523	KIF11	kinesin family member 11	0.84419238
NR_003922	PMCHL2	pro-melanin-concentrating hormone-like 2	0.84352333
NR_003922	PMCHL2	pro-melanin-concentrating hormone-like 2	0.84344384
AB209021	EIF4A2	eukaryotic translation initiation factor 4A, isoform 2	0.84158224
NM_052860	ZNF300	zinc finger protein 300	0.83984044
NM_006820	IFI44L	interferon-induced protein 44-like	0.83860225
NR_001296	TRY6	trypsinogen C	0.83786592
NM_152574	TTC39B	tetratricopeptide repeat domain 39B	0.83616224
NM_003629	PIK3R3	phosphoinositide-3-kinase, regulatory subunit 3 (gamma)	0.83158367
NM_006252	PRKAA2	protein kinase, AMP-activated, alpha 2 catalytic subunit	0.82965467
NM_003318	TTK	TTK protein kinase	0.82876457
NM_001859	SLC31A1	solute carrier family 31 (copper transporters), member 1	0.8263801
NM_014791	MELK	maternal embryonic leucine zipper kinase	0.82473589
NM_002703	PPAT	phosphoribosyl pyrophosphate amidotransferase	0.82440676
NM_006080	SEMA3A	sema domain, immunoglobulin domain (Ig), short basic domain, secreted, (	0.82410902
NM_004083	DDIT3	DNA-damage-inducible transcript 3	0.82264438
NM_002493	NDUFB6	NADH dehydrogenase (ubiquinone) 1 beta subcomplex, 6, 17kDa	0.82107868
NM_031469	SH3BGRL2	SH3 domain binding glutamic acid-rich protein like 2	0.81805136
NM_005622	ACSM3	acyl-CoA synthetase medium-chain family member 3	0.80567515
NM_013230	CD24	CD24 molecule	0.80054282
NM_001135811	FAM60A	family with sequence similarity 60, member A	0.79943136
NM_022346	NCAPG	non-SMC condensin I complex, subunit G	0.79929796
NM_000328	RPGR	retinitis pigmentosa GTPase regulator	0.79886972
NM_198947	FAM111B	family with sequence similarity 111, member B	0.7962005

NM_003234	TFRC	transferrin receptor (p90, CD71)	0.79527144
AB039791	ARP11	actin-related Arp11	0.79517156
NM_014750	DLGAP5	discs, large (Drosophila) homolog-associated protein 5	0.79462947
NM_001099772	CYP4B1	cytochrome P450, family 4, subfamily B, polypeptide 1	0.79433222
NM_007329	DMBT1	deleted in malignant brain tumors 1	0.79345758
NR_002588	SNORA4	small nucleolar RNA, H/ACA box 4	0.79295231
NM_020922	WNK3	WNK lysine deficient protein kinase 3	0.7916566
NM_018490	LGR4	leucine-rich repeat-containing G protein-coupled receptor 4	0.79150053
NM_024122	APOO	apolipoprotein O	0.78937055
NM_002612	PDK4	pyruvate dehydrogenase kinase, isozyme 4	0.78861261
NM_004335	BST2	bone marrow stromal cell antigen 2	0.78782091
NM_173496	MPP7	membrane protein, palmitoylated 7 (MAGUK p55 subfamily member 7)	0.78374024
NM_152321	ERP27	endoplasmic reticulum protein 27	0.78364788
NM_012153	EHF	ets homologous factor	0.78244982
NM_007231	SLC6A14	solute carrier family 6 (amino acid transporter), member 14	0.78211797
NM_004061	CDH12	cadherin 12, type 2 (N-cadherin 2)	0.78172414
NM_001145319	PLS1	plastin 1 (I isoform)	0.77890112
NM_004362	CLGN	calmegin	0.77749163
NM_003270	TSPAN6	tetraspanin 6	0.77515
BC017473	C2orf43	chromosome 2 open reading frame 43	0.77502668
NM_006733	CENPI	centromere protein I	0.77130216
NM_206953	PRAME	preferentially expressed antigen in melanoma	0.76924625
NM_016057	COPZ1	coatomer protein complex, subunit zeta 1	0.76818557
NM_001548	IFIT1	interferon-induced protein with tetratricopeptide repeats 1	0.76800278
NM_000745	CHRNA5	cholinergic receptor, nicotinic, alpha 5	0.76436212
NM_024817	THSD4	thrombospondin, type I, domain containing 4	0.76135002
NM_001017438	CT45A6	cancer/testis antigen family 45, member A6	0.75489736
NM_017645	HAUS6	HAUS augmin-like complex, subunit 6	0.75010539
NM_024491	CEP70	centrosomal protein 70kDa	0.74985541
NM_022662	ANAPC1	anaphase promoting complex subunit 1	0.74875189
NM_014999	RAB21	RAB21, member RAS oncogene family	0.74787747
NM_002483	CEACAM6	carcinoembryonic antigen-related cell adhesion molecule 6 (non-specific cr	0.74707009
NM_006745	SC4MOL	sterol-C4-methyl oxidase-like	0.74703215
NM_002354	EPCAM	epithelial cell adhesion molecule	0.74505219
NM_001099676	C12orf56	chromosome 12 open reading frame 56	0.74330821
NM_001040624	NCALD	neurocalcin delta	0.74253409
NM_001099	ACPP	acid phosphatase, prostate	0.7419994
NM_001113378	FANCI	Fanconi anemia, complementation group I	0.7413188
NM_181806	AASDH	aminoadipate-semialdehyde dehydrogenase	0.74079707
NM_017645	HAUS6	HAUS augmin-like complex, subunit 6	0.73953806
NM_178824	WDR49	WD repeat domain 49	0.73845038
NM_000240	MAOA	monoamine oxidase A	0.73785664
NM_032368	LZIC	leucine zipper and CTNNBIP1 domain containing	0.73668675
NM_014518	ZNF229	zinc finger protein 229	0.73546155
NM_001130157	MLF1	myeloid leukemia factor 1	0.73324582
NM_001017436	CT45A4	cancer/testis antigen family 45, member A4	0.73275261
NM_001039844	ACBD7	acyl-Coenzyme A binding domain containing 7	0.73176291

NM_018431	DOK5	docking protein 5	0.72858539
NM_152582	CT45A2	cancer/testis antigen family 45, member A2	0.72825072
NM_000266	NDP	Norrie disease (pseudoglioma)	0.72715227
NM_004701	CCNB2	cyclin B2	0.72624423
NM_014783	ARHGAP11A	Rho GTPase activating protein 11A	0.72550171
NM_001074	UGT2B7	UDP glucuronosyltransferase 2 family, polypeptide B7	0.72548512
NM_207015	NAALADL2	N-acetylated alpha-linked acidic dipeptidase-like 2	0.72538407
NM_017412	FZD3	frizzled homolog 3 (Drosophila)	0.72476394
NM_032796	SYAP1	synapse associated protein 1, SAP47 homolog (Drosophila)	0.72059021
NM_002125	HLA-DRB5	major histocompatibility complex, class II, DR beta 5	0.71885688
NM_015225	PRUNE2	prune homolog 2 (Drosophila)	0.71814814
NR_002433	SNORD12C	small nucleolar RNA, C/D box 12C	0.71685002
NM_012062	DNM1L	dynamin 1-like	0.71643004
NM_139248	LIPH	lipase, member H	0.71470858
NM_004616	TSPAN8	tetraspanin 8	0.71333595
NM_017996	DET1	de-etiolated homolog 1 (Arabidopsis)	0.71313647
NM_033505	SELI	selenoprotein I	0.70957949
NM_001007551	CT45A5	cancer/testis antigen family 45, member A5	0.70944498
NM_005447	RASSF9	Ras association (RalGDS/AF-6) domain family (N-terminal) member 9	0.70944102
NM_017697	ESRP1	epithelial splicing regulatory protein 1	0.70943792
NM_178815	ARL5B	ADP-ribosylation factor-like 5B	0.7088004
NM_001005353	AK3L1	adenylate kinase 3-like 1	0.70876562
NM_152259	C15orf42	chromosome 15 open reading frame 42	0.7054673
NM_002637	PHKA1	phosphorylase kinase, alpha 1 (muscle)	0.70463684
NM_012137	DDAH1	dimethylarginine dimethylaminohydrolase 1	0.70456418
NM_001134296	AP3M2	adaptor-related protein complex 3, mu 2 subunit	0.70389932
NM_002164	IDO1	indoleamine 2,3-dioxygenase 1	0.70365679
NM_001565	CXCL10	chemokine (C-X-C motif) ligand 10	0.70033133
NM_002849	PTPRR	protein tyrosine phosphatase, receptor type, R	0.69841442
NM_006639	CYSLTR1	cysteinyl leukotriene receptor 1	0.69813924
NM_022662	ANAPC1	anaphase promoting complex subunit 1	0.69801882
NM_152697	SLC44A5	solute carrier family 44, member 5	0.69761002
NM_004484	GPC3	glypican 3	0.69527378
NM_001398	ECH1	enoyl Coenzyme A hydratase 1, peroxisomal	0.69422543
NM_007235	XPOT	exportin, tRNA (nuclear export receptor for tRNAs)	0.69355512
NM_003477	PDHX	pyruvate dehydrogenase complex, component X	0.69159384
NM_138573	NRG4	neuregulin 4	0.69147562
NM_003522	HIST1H2BF	histone cluster 1, H2bf	0.69129073
NM_001146108	PTGR1	prostaglandin reductase 1	0.69038632
NM_001105243	PCDH19	protocadherin 19	0.68981733
NM_173567	EPHX4	epoxide hydrolase 4	0.68967232
NR_002563	SNORD27	small nucleolar RNA, C/D box 27	0.68600932
NM_005013	NUCB2	nucleobindin 2	0.68555499
NM_001130963	TMEM194A	transmembrane protein 194A	0.684286
NM_182751	MCM10	minichromosome maintenance complex component 10	0.67886761
NM_000338	SLC12A1	solute carrier family 12 (sodium/potassium/chloride transporters), member	0.67565657
NM_002759	EIF2AK2	eukaryotic translation initiation factor 2-alpha kinase 2	0.67457751

NM_080657	RSAD2	radical S-adenosyl methionine domain containing 2	0.67316269
NM_001042519	C2orf88	chromosome 2 open reading frame 88	0.67211375
NM_016323	HERC5	hect domain and RLD 5	0.67138965
NM_031217	KIF18A	kinesin family member 18A	0.67011376
NM_001145191	LOC285550	hypothetical protein LOC285550	0.67007598
NM_002631	PGD	phosphogluconate dehydrogenase	0.66770315
NM_021252	RAB18	RAB18, member RAS oncogene family	0.66767421
NM_005530	IDH3A	isocitrate dehydrogenase 3 (NAD+) alpha	0.66755188
NM_018269	ADI1	acireductone dioxygenase 1	0.66691515
NM_004293	GDA	guanine deaminase	0.66569702
NM_001136262	LOC552889	hypothetical protein LOC552889	0.66330936
NM_001039569	AP1S3	adaptor-related protein complex 1, sigma 3 subunit	0.66235686
BC105048	KIAA1958	KIAA1958	0.66205121
NM_001135694	VDAC3	voltage-dependent anion channel 3	0.66164586
NM_032299	DCUN1D5	DCN1, defective in cullin neddylation 1, domain containing 5 (S. cerevisiae)	0.66135362
NM_002770	PRSS2	protease, serine, 2 (trypsin 2)	0.66002368
NM_022780	RMND5A	required for meiotic nuclear division 5 homolog A (S. cerevisiae)	0.65999062
NM_000933	PLCB4	phospholipase C, beta 4	0.65956939
NM_016570	ERGIC2	ERGIC and golgi 2	0.65805238
NM_015065	EXPH5	exophilin 5	0.65695095
NM_012415	RAD54B	RAD54 homolog B (S. cerevisiae)	0.65653189
BC008502	C4orf34	chromosome 4 open reading frame 34	0.65450685
NM_024838	THNSL1	threonine synthase-like 1 (S. cerevisiae)	0.65430472
NM_003155	STC1	stanniocalcin 1	0.65292591
NM_006918	SC5DL	sterol-C5-desaturase (ERG3 delta-5-desaturase homolog, S. cerevisiae)-like	0.65276161
NM_004697	PRPF4	PRP4 pre-mRNA processing factor 4 homolog (yeast)	0.6523723
NM_181783	TMTC3	transmembrane and tetratricopeptide repeat containing 3	0.65090729
NM_002535	OAS2	2'-5'-oligoadenylate synthetase 2, 69/71kDa	0.65087636
NM_020401	NUP107	nucleoporin 107kDa	0.65054221
NM_182758	WDR72	WD repeat domain 72	0.64970718
NM_024604	RPAP3	RNA polymerase II associated protein 3	0.64950608
NM_012431	SEMA3E	sema domain, immunoglobulin domain (Ig), short basic domain, secreted, (	0.6494893
NM_000777	CYP3A5	cytochrome P450, family 3, subfamily A, polypeptide 5	0.64920039
NM_020672	S100A14	S100 calcium binding protein A14	0.64690009
NM_175859	CTPS2	CTP synthase II	0.64574938
NM_005047	PSMD5	proteasome (prosome, macropain) 26S subunit, non-ATPase, 5	0.64504596
NM_004999	MYO6	myosin VI	0.64450603
NM_001873	CPE	carboxypeptidase E	0.64396658
NM_001114120	DEPDC1	DEP domain containing 1	0.64344222
NM_138374	ZNF845	zinc finger protein 845	0.64252445
NR_024333	LOC147727	hypothetical LOC147727	0.64079215
NM_020242	KIF15	kinesin family member 15	0.64037362
NM_152282	ACPL2	acid phosphatase-like 2	0.64026782
NM_003215	TEC	tec protein tyrosine kinase	0.64004697
NM_006089	SCML2	sex comb on midleg-like 2 (Drosophila)	0.63889701
NM_022374	ATL2	atlastin GTPase 2	0.63875316
NM_006227	PLTP	phospholipid transfer protein	0.63868195

NM_018365	MNS1	meiosis-specific nuclear structural 1	0.63860673
NM_198433	AURKA	aurora kinase A	0.63834046
NM_006898	HOXD3	homeobox D3	0.63788683
NM_178566	ZDHHC21	zinc finger, DHHC-type containing 21	0.63761861
NM_021244	RRAGD	Ras-related GTP binding D	0.63658999
NM_005983	SKP2	S-phase kinase-associated protein 2 (p45)	0.63615113
NM_017631	DDX60	DEAD (Asp-Glu-Ala-Asp) box polypeptide 60	0.63563172
NM_006408	AGR2	anterior gradient homolog 2 (Xenopus laevis)	0.63562756
NM_198150	ARSK	arylsulfatase family, member K	0.6352923
NM_173842	IL1RN	interleukin 1 receptor antagonist	0.63514371
NM_001185	AZGP1	alpha-2-glycoprotein 1, zinc-binding	0.63501863
NM_014736	KIAA0101	KIAA0101	0.63444991
NM_003611	OFD1	oral-facial-digital syndrome 1	0.63444192
NM_032730	RTN4IP1	reticulon 4 interacting protein 1	0.63400501
NM_017741	C4orf30	chromosome 4 open reading frame 30	0.63390824
NM_018356	C5orf22	chromosome 5 open reading frame 22	0.63379448
NM_001145065	KIAA1680	KIAA1680 protein	0.63252582
NM_013277	RACGAP1	Rac GTPase activating protein 1	0.63249377
NM_012420	IFIT5	interferon-induced protein with tetratricopeptide repeats 5	0.63242896
NM_017641	KIF21A	kinesin family member 21A	0.63226836
NM_016824	ADD3	adducin 3 (gamma)	0.63148947
NM_001100880	ST20	suppressor of tumorigenicity 20	0.63129739
NM_018843	SLC25A40	solute carrier family 25, member 40	0.6300016
NM_033495	KLHL13	kelch-like 13 (Drosophila)	0.62892193
NM_181716	CENPV	centromere protein V	0.62884162
NM_006933	SLC5A3	solute carrier family 5 (sodium/myo-inositol cotransporter), member 3	0.62875059
NM_000888	ITGB6	integrin, beta 6	0.62708327
NM_145290	GPR125	G protein-coupled receptor 125	0.62693833
NM_003981	PRC1	protein regulator of cytokinesis 1	0.62651049
NM_005409	CXCL11	chemokine (C-X-C motif) ligand 11	0.62564287
NM_001145775	FKBP5	FK506 binding protein 5	0.62411041
NM_002301	LDHC	lactate dehydrogenase C	0.62408152
NM_021190	PTBP2	polypyrimidine tract binding protein 2	0.62350456
NM_003196	TCEA3	transcription elongation factor A (SII), 3	0.62342018
NM_024670	SUV39H2	suppressor of variegation 3-9 homolog 2 (Drosophila)	0.62249888
NM_001296	CCBP2	chemokine binding protein 2	0.62134982
NM_001017417	CT45A1	cancer/testis antigen family 45, member A1	0.62098613
NM_001127698	SPINK5	serine peptidase inhibitor, Kazal type 5	0.61994667
NM_033427	CTTNBP2	cortactin binding protein 2	0.61916141
NM_001159642	BNIPL	BCL2/adenovirus E1B 19kD interacting protein like	0.61903119
NM_022090	C5orf54	chromosome 5 open reading frame 54	0.6190049
NM_024896	ERMP1	endoplasmic reticulum metallopeptidase 1	0.61872724
NM_001001411	ZNF676	zinc finger protein 676	0.61872221
NM_001099669	HIGD1A	HIG1 hypoxia inducible domain family, member 1A	0.61860304
NM_005687	FARSB	phenylalanyl-tRNA synthetase, beta subunit	0.61767278
NM_015938	NMD3	NMD3 homolog (S. cerevisiae)	0.61619522
NM_005333	HCCS	holocytochrome c synthase (cytochrome c heme-lyase)	0.61580201

NM_032844	MASTL	microtubule associated serine/threonine kinase-like	0.61520732
NM_080652	TMEM41A	transmembrane protein 41A	0.61500288
NM_005325	HIST1H1A	histone cluster 1, H1a	0.61438115
NM_001884	HAPLN1	hyaluronan and proteoglycan link protein 1	0.61436466
NM_000946	PRIM1	primase, DNA, polypeptide 1 (49kDa)	0.61413698
NM_012259	HEY2	hairy/enhancer-of-split related with YRPW motif 2	0.61408086
NM_018448	CAND1	cullin-associated and neddylation-dissociated 1	0.61345999
NM_144973	DENND5B	DENN/MADD domain containing 5B	0.61332752
NM_153618	SEMA6D	sema domain, transmembrane domain (TM), and cytoplasmic domain, (sem	0.61324997
NM_005564	LCN2	lipocalin 2	0.61265672
NM_020675	SPC25	SPC25, NDC80 kinetochore complex component, homolog (S. cerevisiae)	0.61248662
NM_001918	DBT	dihydrolipoamide branched chain transacylase E2	0.6122989
NM_024511	HAUS3	HAUS augmin-like complex, subunit 3	0.61202781
NM_014735	PHF16	PHD finger protein 16	0.61141042
NM_017439	PION	pigeon homolog (Drosophila)	0.61132969
NM_033360	KRAS	v-Ki-ras2 Kirsten rat sarcoma viral oncogene homolog	0.61024858
BC014881	C9orf80	chromosome 9 open reading frame 80	0.60938329
NM_024621	VEPH1	ventricular zone expressed PH domain homolog 1 (zebrafish)	0.60898978
NM_014695	CCDC144A	coiled-coil domain containing 144A	0.60823797
NM_006601	PTGES3	prostaglandin E synthase 3 (cytosolic)	0.60791959
NR_027471	LOC440173	hypothetical LOC440173	0.60765919
NM_019091	PLEKHA3	pleckstrin homology domain containing, family A (phosphoinositide bindin	0.6075081
NM_016073	HDGFRP3	hepatoma-derived growth factor, related protein 3	0.60671932
NM_020909	EPB41L5	erythrocyte membrane protein band 4.1 like 5	0.60638414
NM_001080449	DNA2	DNA replication helicase 2 homolog (yeast)	0.60617602
NM_032689	ZNF607	zinc finger protein 607	0.60585955
NM_001048	SST	somatostatin	0.60426582
NM_014398	LAMP3	lysosomal-associated membrane protein 3	0.60358673
NM_001423	EMP1	epithelial membrane protein 1	0.6034739
NM_001142292	LMAN2L	lectin, mannose-binding 2-like	0.60339395
NM_021067	GINS1	GINS complex subunit 1 (Psf1 homolog)	0.60175877
NM_021129	PPA1	pyrophosphatase (inorganic) 1	0.60164538
NM_003328	TXK	TXK tyrosine kinase	0.59991676
NM_001037540	SCML1	sex comb on midleg-like 1 (Drosophila)	0.5998589
NM_001099668	HIGD1A	HIG1 hypoxia inducible domain family, member 1A	0.59982468
NM_001113528	METT5D1	methyltransferase 5 domain containing 1	0.59954521
ENST00000313266	LOC100129455	hypothetical LOC100129455	0.59903699
NM_001013625	C1orf192	chromosome 1 open reading frame 192	0.59898253
NM_014554	SENP1	SUMO1/sentrin specific peptidase 1	0.59891301
NM_003137	SRPK1	SFRS protein kinase 1	0.59841645
NM_002499	NEO1	neogenin homolog 1 (chicken)	0.59758778
NM_019053	EXOC6	exocyst complex component 6	0.59700026
NM_058170	OLFM3	olfactomedin 3	0.59666972
NM_014050	MRPL42	mitochondrial ribosomal protein L42	0.5956027
NM_005180	BMI1	BMI1 polycomb ring finger oncogene	0.59440573
NM_006530	YEATS4	YEATS domain containing 4	0.59433492
NM_018427	RRN3	RRN3 RNA polymerase I transcription factor homolog (S. cerevisiae)	0.59429819

NM_015895	GMNN	geminin, DNA replication inhibitor	0.59387548
NM_006209	ENPP2	ectonucleotide pyrophosphatase/phosphodiesterase 2	0.59301402
NM_020466	LYRM2	LYR motif containing 2	0.59292036
NM_004136	IREB2	iron-responsive element binding protein 2	0.5926095
NM_004438	EPHA4	EPH receptor A4	0.59260896
NM_052879	LARP4	La ribonucleoprotein domain family, member 4	0.59259855
NM_006166	NFYB	nuclear transcription factor Y, beta	0.59255458
NM_006417	IFI44	interferon-induced protein 44	0.59232875
NM_018098	ECT2	epithelial cell transforming sequence 2 oncogene	0.59216553
NM_006345	SLC30A9	solute carrier family 30 (zinc transporter), member 9	0.59172064
NM_014016	SACM1L	SAC1 suppressor of actin mutations 1-like (yeast)	0.59135432
NM_133646	ZAK	sterile alpha motif and leucine zipper containing kinase AZK	0.59088628
AY956764	HSP90AB3P	heat shock protein 90kDa alpha (cytosolic), class B member 3 (pseudogene	0.59086035
NM_152376	UBXN10	UBX domain protein 10	0.59029391
NM_000170	GLDC	glycine dehydrogenase (decarboxylating)	0.58984172
NM_001004470	ST8SIA6	ST8 alpha-N-acetyl-neuraminide alpha-2,8-sialyltransferase 6	0.58969858
NM_005653	TFCP2	transcription factor CP2	0.58965019
NM_002462	MX1	myxovirus (influenza virus) resistance 1, interferon-inducible protein p78 (m	0.58673264
NM_001866	COX7B	cytochrome c oxidase subunit VIIb	0.58634424
NM_005153	USP10	ubiquitin specific peptidase 10	0.58561938
NM_021627	SENP2	SUMO1/sentrin/SMT3 specific peptidase 2	0.58508281
NM_014729	TOX	thymocyte selection-associated high mobility group box	0.58491723
ENST00000322630	TIMP2	TIMP metallopeptidase inhibitor 2	-0.5844229
NM_002309	LIF	leukemia inhibitory factor (cholinergic differentiation factor)	-0.5844452
NM_033178	DUX4	double homeobox, 4	-0.5845739
NM_001111307	PDE4A	phosphodiesterase 4A, cAMP-specific (phosphodiesterase E2 dunce homolo	-0.5853093
NM_006312	NCOR2	nuclear receptor co-repressor 2	-0.5857246
NM_003246	THBS1	thrombospondin 1	-0.586849
NM_014280	DNAJC8	DnaJ (Hsp40) homolog, subfamily C, member 8	-0.5872278
NR_003664	SPDYE8P	speedy homolog E8 (Xenopus laevis), pseudogene	-0.5888803
NR_003664	SPDYE8P	speedy homolog E8 (Xenopus laevis), pseudogene	-0.5888803
NM_014705	DOCK4	dedicator of cytokinesis 4	-0.5890887
NR_003260	C15orf51	chromosome 15 open reading frame 51	-0.5894864
NM_001105662	USP17	ubiquitin specific peptidase 17	-0.5898291
NM_175064	SPDYE1	speedy homolog E1 (Xenopus laevis)	-0.590802
NM_022748	TNS3	tensin 3	-0.5910719
NM_002036	DARC	Duffy blood group, chemokine receptor	-0.591231
NM_001017995	SH3PXD2B	SH3 and PX domains 2B	-0.5920958
NM_032208	ANTXR1	anthrax toxin receptor 1	-0.593211
NM_004978	KCNC4	potassium voltage-gated channel, Shaw-related subfamily, member 4	-0.5938066
NM_015892	GALNAC4S-6ST	B cell RAG associated protein	-0.5951224
NM_175064	SPDYE1	speedy homolog E1 (Xenopus laevis)	-0.5955354
NM_003255	TIMP2	TIMP metallopeptidase inhibitor 2	-0.5959109
NM_001040084	ANXA8	annexin A8	-0.5970847
NM_001458	FLNC	filamin C, gamma (actin binding protein 280)	-0.597122
NM_001105662	USP17	ubiquitin specific peptidase 17	-0.5973274
NM_001105662	USP17	ubiquitin specific peptidase 17	-0.5973274

NM_001105662	USP17	ubiquitin specific peptidase 17	-0.5973274
NM_001105662	USP17	ubiquitin specific peptidase 17	-0.5973274
NM_001105662	USP17	ubiquitin specific peptidase 17	-0.5973274
NM_001105662	USP17	ubiquitin specific peptidase 17	-0.5973274
NM_001105662	USP17	ubiquitin specific peptidase 17	-0.5973274
NR_003320 // NI	SNRPN // SNOR	small nuclear ribonucleoprotein polypeptide N // small nucleolar RNA, C/I	-0.5977604
NR_003320 // NI	SNRPN // SNOR	small nuclear ribonucleoprotein polypeptide N // small nucleolar RNA, C/I	-0.5977604
NM_153184	CADM2	cell adhesion molecule 2	-0.5983402
NM_001127392	C11orf9	chromosome 11 open reading frame 9	-0.5991297
NM_006291	TNFAIP2	tumor necrosis factor, alpha-induced protein 2	-0.6018172
NR_002326	SNORA64	small nucleolar RNA, H/ACA box 64	-0.6024483
NM_001893	CSNK1D	casein kinase 1, delta	-0.6029423
NM_003786	ABCC3	ATP-binding cassette, sub-family C (CFTR/MRP), member 3	-0.6061022
NR_003664	SPDYE8P	speedy homolog E8 (Xenopus laevis), pseudogene	-0.6094706
NM_003062	SLIT3	slit homolog 3 (Drosophila)	-0.6100206
NM_001129	AEBP1	AE binding protein 1	-0.6101724
NR_003008	SCARNA5	small Cajal body-specific RNA 5	-0.6112996
NM_002742	PRKD1	protein kinase D1	-0.6114181
NR_003664	SPDYE8P	speedy homolog E8 (Xenopus laevis), pseudogene	-0.611498
NM_001797	CDH11	cadherin 11, type 2, OB-cadherin (osteoblast)	-0.6120371
NM_201433	GAS7	growth arrest-specific 7	-0.6126388
NM_052917	GALNT13	UDP-N-acetyl-alpha-D-galactosamine:polypeptide N-acetylgalactosaminyltr	-0.6141818
NM_005841	SPRY1	sprouty homolog 1, antagonist of FGF signaling (Drosophila)	-0.6149936
NR_027458	SYT14	synaptotagmin XIV	-0.6152704
NM_001080495	TNRC18	trinucleotide repeat containing 18	-0.6156978
NM_001098845	ANXA8L1	annexin A8-like 1	-0.6161178
NM_001630	ANXA8L2	annexin A8-like 2	-0.6162252
NM_001008271	SCXA	scleraxis homolog A (mouse)	-0.617417
NM_001008271	SCXA	scleraxis homolog A (mouse)	-0.617417
NM_001099435	SPDYE5	speedy homolog E5 (Xenopus laevis)	-0.6186803
NM_007332	TRPA1	transient receptor potential cation channel, subfamily A, member 1	-0.6191273
NR_024344	LOC283174	hypothetical LOC283174	-0.6192804
AF495523	REXO1L1	REX1, RNA exonuclease 1 homolog (S. cerevisiae)-like 1	-0.6194277
NM_002663	PLD2	phospholipase D2	-0.6201861
NM_178831	GATS	GATS, stromal antigen 3 opposite strand	-0.6204409
NM_004105	EFEMP1	EGF-containing fibulin-like extracellular matrix protein 1	-0.6227285
NM_198098	AQP1	aquaporin 1 (Colton blood group)	-0.6240028
NM_000612	IGF2	insulin-like growth factor 2 (somatomedin A)	-0.6241641
NM_014957	DENND3	DENN/MADD domain containing 3	-0.624646
NR_003666	SPDYE7P	speedy homolog E7 (Xenopus laevis), pseudogene	-0.6250371
NM_080826	ISM1	isthmin 1 homolog (zebrafish)	-0.6250879
NM_005940	MMP11	matrix metallopeptidase 11 (stromelysin 3)	-0.6252288
NM_001136273	ZFP92	zinc finger protein 92 homolog (mouse)	-0.6256978
NM_178860	SEZ6	seizure related 6 homolog (mouse)	-0.6262402
NM_017551	GRID1	glutamate receptor, ionotropic, delta 1	-0.6275046
NM_138689 // N	PPP1R14B // PPI	protein phosphatase 1, regulatory (inhibitor) subunit 14B // protein phosp	-0.628357
NM_030569	ITIH5	inter-alpha (globulin) inhibitor H5	-0.6323996

NM_001014448	CPZ	carboxypeptidase Z	-0.6330013
NM_001079807	PGA3	pepsinogen 3, group I (pepsinogen A)	-0.6331295
NM_001717	BNC1	basonuclin 1	-0.6343671
NR_003664	SPDYE8P	speedy homolog E8 (Xenopus laevis), pseudogene	-0.6345874
NM_139072	DNER	delta/notch-like EGF repeat containing	-0.634937
NM_004205	USP2	ubiquitin specific peptidase 2	-0.635984
NM_001854	COL11A1	collagen, type XI, alpha 1	-0.6372748
NM_153444	OR5P2	olfactory receptor, family 5, subfamily P, member 2	-0.6373822
NM_004058	CAPS	calcyphosine	-0.6377526
NR_003360 // NI	SNRPN // SNOR	small nuclear ribonucleoprotein polypeptide N // small nucleolar RNA, C/I	-0.6379715
NR_003303 // NI	SNRPN // SNOR	small nuclear ribonucleoprotein polypeptide N // small nucleolar RNA, C/I	-0.6381053
NR_003664	SPDYE8P	speedy homolog E8 (Xenopus laevis), pseudogene	-0.6398466
NM_001079529	FAM153B	family with sequence similarity 153, member B	-0.6409069
NM_000396	CTSK	cathepsin K	-0.6415802
NM_006650	CPLX2	complexin 2	-0.6423659
NM_012242	DKK1	dickkopf homolog 1 (Xenopus laevis)	-0.6429116
NM_000362	TIMP3	TIMP metallopeptidase inhibitor 3	-0.6432878
NM_002615	SERPINF1	serpin peptidase inhibitor, clade F (alpha-2 antiplasmin, pigment epitheliun	-0.6436957
NR_015377	LOC654433	hypothetical LOC654433	-0.644179
NR_003260	C15orf51	chromosome 15 open reading frame 51	-0.6447195
NR_003260	C15orf51	chromosome 15 open reading frame 51	-0.6447195
NR_003260	C15orf51	chromosome 15 open reading frame 51	-0.6447195
NM_001843	CNTN1	contactin 1	-0.6447802
NM_001080495	TNRC18	trinucleotide repeat containing 18	-0.6462308
NM_001831	CLU	clusterin	-0.6467358
NM_133373	PLCD3	phospholipase C, delta 3	-0.6474533
NM_000855	GUCY1A2	guanylate cyclase 1, soluble, alpha 2	-0.6480953
NM_053056	CCND1	cyclin D1	-0.6481172
NM_153366	SVEP1	sushi, von Willebrand factor type A, EGF and pentraxin domain containing	-0.6485452
NR_003664	SPDYE8P	speedy homolog E8 (Xenopus laevis), pseudogene	-0.6504223
NM_000358	TGFBI	transforming growth factor, beta-induced, 68kDa	-0.6509127
NM_207380	C15orf52	chromosome 15 open reading frame 52	-0.6513055
AF043897	C9orf3	chromosome 9 open reading frame 3	-0.6513919
BC033223	SLC45A4	solute carrier family 45, member 4	-0.6513957
NM_003033	ST3GAL1	ST3 beta-galactoside alpha-2,3-sialyltransferase 1	-0.6536423
NR_003594	REXO1L2P	REX1, RNA exonuclease 1 homolog (S. cerevisiae)-like 2 (pseudogene)	-0.6541288
NM_182915	STEAP3	STEAP family member 3	-0.6541729
NR_003664	SPDYE8P	speedy homolog E8 (Xenopus laevis), pseudogene	-0.6546172
NM_032415	CARD11	caspase recruitment domain family, member 11	-0.6546453
NM_001004439	ITGA11	integrin, alpha 11	-0.6547366
NM_018689	KIAA1199	KIAA1199	-0.6552314
NM_000693	ALDH1A3	aldehyde dehydrogenase 1 family, member A3	-0.6559405
NM_033178	DUX4	double homeobox, 4	-0.6576861
NR_002312	RPPH1	ribonuclease P RNA component H1	-0.6581534
NM_006475	POSTN	periostin, osteoblast specific factor	-0.6585756
NM_003966	SEMA5A	sema domain, seven thrombospondin repeats (type 1 and type 1-like), tran	-0.6586299
NM_014987	IGSF9B	immunoglobulin superfamily, member 9B	-0.6586531

NR_003318 // NI	SNRPN // SNOR	small nuclear ribonucleoprotein polypeptide N // small nucleolar RNA, C/I	-0.6589514
NR_003318 // NI	SNRPN // SNOR	small nuclear ribonucleoprotein polypeptide N // small nucleolar RNA, C/I	-0.6589514
NM_002345	LUM	lumican	-0.6600371
NM_033178	DUX4	double homeobox, 4	-0.6605116
NM_033178	DUX4	double homeobox, 4	-0.6605116
NM_033178	DUX4	double homeobox, 4	-0.6605116
NM_033178	DUX4	double homeobox, 4	-0.6605116
NM_033178	DUX4	double homeobox, 4	-0.6605116
NM_015039	NMNAT2	nicotinamide nucleotide adenylyltransferase 2	-0.6640617
NM_002343	LTF	lactotransferrin	-0.6651012
NR_003316 // NI	SNRPN // SNOR	small nuclear ribonucleoprotein polypeptide N // small nucleolar RNA, C/I	-0.6655746
NM_033178	DUX4	double homeobox, 4	-0.665603
NM_033178	DUX4	double homeobox, 4	-0.665603
NM_001079527	FAM153C	family with sequence similarity 153, member C	-0.6677946
NR_003594	REXO1L2P	REX1, RNA exonuclease 1 homolog (S. cerevisiae)-like 2 (pseudogene)	-0.6687969
NR_003594	REXO1L2P	REX1, RNA exonuclease 1 homolog (S. cerevisiae)-like 2 (pseudogene)	-0.6687969
NR_003594	REXO1L2P	REX1, RNA exonuclease 1 homolog (S. cerevisiae)-like 2 (pseudogene)	-0.6687969
NM_000916	OXTR	oxytocin receptor	-0.6693285
NR_003594	REXO1L2P	REX1, RNA exonuclease 1 homolog (S. cerevisiae)-like 2 (pseudogene)	-0.6713772
NM_022489	INF2	inverted formin, FH2 and WH2 domain containing	-0.6718609
NM_004956	ETV1	ets variant 1	-0.6732543
NM_032532	FNDC1	fibronectin type III domain containing 1	-0.6742197
NM_002084	GPX3	glutathione peroxidase 3 (plasma)	-0.6757626
NM_001276	CHI3L1	chitinase 3-like 1 (cartilage glycoprotein-39)	-0.6764738
NM_001080421	UNC13A	unc-13 homolog A (C. elegans)	-0.6803985
AK131514	FLJ16734	hypothetical LOC641928	-0.6814658
AK131040	LOC388022	hypothetical gene supported by AK131040	-0.681996
NM_172239	REXO1L1	REX1, RNA exonuclease 1 homolog (S. cerevisiae)-like 1	-0.6821051
NM_005576	LOXL1	lysyl oxidase-like 1	-0.6826869
NM_198465	NRK	Nik related kinase	-0.6831147
NM_001079808	PGA4	pepsinogen 4, group I (pepsinogen A)	-0.6831424
NM_002048	GAS1	growth arrest-specific 1	-0.6850627
BC110394	IGKC	immunoglobulin kappa constant	-0.6859098
NM_002146	HOXB3	homeobox B3	-0.6904289
NM_002010	FGF9	fibroblast growth factor 9 (glia-activating factor)	-0.692203
NM_015717	CD207	CD207 molecule, langerin	-0.6928237
NM_144633	KCNH8	potassium voltage-gated channel, subfamily H (eag-related), member 8	-0.6958689
NR_002962	SNORA23	small nucleolar RNA, H/ACA box 23	-0.6959718
NM_030893	CD1E	CD1e molecule	-0.6989136
NM_003247	THBS2	thrombospondin 2	-0.6994376
AK125737 // AK1	LOC440570 // L(	hypothetical LOC440570 // hypothetical LOC440570	-0.701761
AK125737 // AK1	LOC440570 // L(	hypothetical LOC440570 // hypothetical LOC440570	-0.701761
NM_030761	WNT4	wingless-type MMTV integration site family, member 4	-0.7022689
NM_001031618	SPDYE2	speedy homolog E2 (Xenopus laevis)	-0.7035849
NM_001003802	SMARCD3	SWI/SNF related, matrix associated, actin dependent regulator of chromati	-0.7037606
AK302597 // AK3	POM121L8P // F	POM121 membrane glycoprotein-like 8 (rat) pseudogene // POM121 mem	-0.7050085
NM_001202	BMP4	bone morphogenetic protein 4	-0.7121071

NR_003594	REXO1L2P	REX1, RNA exonuclease 1 homolog (S. cerevisiae)-like 2 (pseudogene)	-0.7142775
NM_133369	UNC5A	unc-5 homolog A (C. elegans)	-0.715124
ENST00000390266	LOC651751	similar to Ig kappa chain V-II region RPMI 6410 precursor	-0.7159498
NM_001018111	PODXL	podocalyxin-like	-0.7165068
NM_001146210	SPDYE6	speedy homolog E6 (Xenopus laevis)	-0.7169979
NM_014224	PGA5	pepsinogen 5, group I (pepsinogen A)	-0.7170548
NM_001031618	SPDYE2	speedy homolog E2 (Xenopus laevis)	-0.7185056
NM_006952	UPK1B	uroplakin 1B	-0.7201784
NM_002705	PPL	periplakin	-0.7208058
NM_001765	CD1C	CD1c molecule	-0.7245743
NM_138420	AHNAK2	AHNAK nucleoprotein 2	-0.7274591
NM_002501	NFIX	nuclear factor I/X (CCAAT-binding transcription factor)	-0.7309073
NM_004530	MMP2	matrix metallopeptidase 2 (gelatinase A, 72kDa gelatinase, 72kDa type IV c	-0.7331163
NM_001711	BGN	biglycan	-0.7336677
NM_005556	KRT7	keratin 7	-0.7364452
NM_022127	SLC28A3	solute carrier family 28 (sodium-coupled nucleoside transporter), member 3	-0.7413985
NM_024794	EPHX3	epoxide hydrolase 3	-0.7414571
AF043897	C9orf3	chromosome 9 open reading frame 3	-0.7420272
NM_033178	DUX4	double homeobox, 4	-0.7451033
NM_001017915	INPP5D	inositol polyphosphate-5-phosphatase, 145kDa	-0.7469917
NM_005545	ISLR	immunoglobulin superfamily containing leucine-rich repeat	-0.7512967
---	---	---	-0.7522138
NM_001763	CD1A	CD1a molecule	-0.7542769
---	---	---	-0.7566687
NM_004445	EPHB6	EPH receptor B6	-0.756673
NM_022731	NUCKS1	nuclear casein kinase and cyclin-dependent kinase substrate 1	-0.7577432
NM_022475	HHIP	hedgehog interacting protein	-0.7590401
NM_139055	ADAMTS15	ADAM metallopeptidase with thrombospondin type 1 motif, 15	-0.7616786
NM_024721	ZFHX4	zinc finger homeobox 4	-0.7620738
NM_001080495	TNRC18	trinucleotide repeat containing 18	-0.7624972
NM_005937	MLLT6	myeloid/lymphoid or mixed-lineage leukemia (trithorax homolog, Drosoph	-0.7637821
NM_016240	SCARA3	scavenger receptor class A, member 3	-0.7714789
NM_003749	IRS2	insulin receptor substrate 2	-0.7720224
NM_001447	FAT2	FAT tumor suppressor homolog 2 (Drosophila)	-0.775485
NM_000428	LTBP2	latent transforming growth factor beta binding protein 2	-0.7755655
BC132953	C8orf15	chromosome 8 open reading frame 15	-0.7783029
NR_003594	REXO1L2P	REX1, RNA exonuclease 1 homolog (S. cerevisiae)-like 2 (pseudogene)	-0.7814956
NM_024690	MUC16	mucin 16, cell surface associated	-0.7857184
NM_000956	PTGER2	prostaglandin E receptor 2 (subtype EP2), 53kDa	-0.7906596
NM_001079529	FAM153B	family with sequence similarity 153, member B	-0.7913675
NM_033178	DUX4	double homeobox, 4	-0.7987266
---	---	---	-0.7991984
NR_027279	DUB4	deubiquitinating enzyme DUB4	-0.7995314
---	---	---	-0.8049433
BC093097	IGKC	immunoglobulin kappa constant	-0.807217
NM_003654	CHST1	carbohydrate (keratan sulfate Gal-6) sulfotransferase 1	-0.8079668
---	---	---	-0.8096149

NM_000820	GAS6	growth arrest-specific 6	-0.8115258
NM_001128205	SULF1	sulfatase 1	-0.8121844
NM_002658	PLAU	plasminogen activator, urokinase	-0.8128709
NM_001898	CST1	cystatin SN	-0.8176982
NM_004994	MMP9	matrix metallopeptidase 9 (gelatinase B, 92kDa gelatinase, 92kDa type IV c	-0.8193481
NM_014485	PGDS	prostaglandin D2 synthase, hematopoietic	-0.8229312
NM_020223	FAM20C	family with sequence similarity 20, member C	-0.8241042
NR_003323 // NI	SNRPN // SNOR	small nuclear ribonucleoprotein polypeptide N // small nucleolar RNA, C/I	-0.8284582
NM_020733	HEG1	HEG homolog 1 (zebrafish)	-0.8406983
NM_003014	SFRP4	secreted frizzled-related protein 4	-0.8504057
AF043897	C9orf3	chromosome 9 open reading frame 3	-0.8507948
NM_001005463	EBF3	early B-cell factor 3	-0.8527335
---	---	---	-0.8557671
NR_002449	SNORA65	small nucleolar RNA, H/ACA box 65	-0.8580299
NM_001039350	DPP6	dipeptidyl-peptidase 6	-0.860412
NR_003321 // NI	SNRPN // SNOR	small nuclear ribonucleoprotein polypeptide N // small nucleolar RNA, C/I	-0.8645501
NM_001850	COL8A1	collagen, type VIII, alpha 1	-0.8850874
---	---	---	-0.8871185
NM_001145204	LOC729993	hypothetical protein LOC729993	-0.8942506
NM_019601	SUSD2	sushi domain containing 2	-0.8949013
NM_033223	GABRG3	gamma-aminobutyric acid (GABA) A receptor, gamma 3	-0.8960841
---	---	---	-0.9109589
---	---	---	-0.9241143
NM_021101	CLDN1	claudin 1	-0.9252019
NM_001080512	BICC1	bicaudal C homolog 1 (Drosophila)	-0.9253032
NM_058229	FBXO32	F-box protein 32	-0.9374885
---	---	---	-0.9376755
---	---	---	-0.9383524
NM_000095	COMP	cartilage oligomeric matrix protein	-0.9489644
AK092862	FLJ41484	hypothetical LOC650669	-0.9496569
BC018448	MALAT1	metastasis associated lung adenocarcinoma transcript 1 (non-protein codir	-0.9528193
NM_002204	ITGA3	integrin, alpha 3 (antigen CD49C, alpha 3 subunit of VLA-3 receptor)	-0.9565525
NM_002281	KRT81	keratin 81	-0.9572443
NM_001127500	MET	met proto-oncogene (hepatocyte growth factor receptor)	-0.967565
NM_001085423	C17orf60	chromosome 17 open reading frame 60	-0.9789255
NM_000213	ITGB4	integrin, beta 4	-0.9816535
NM_001337	CX3CR1	chemokine (C-X3-C motif) receptor 1	-1.0112926
NM_152611	LRRN4	leucine rich repeat neuronal 4	-1.0164014
NM_181690	AKT3	v-akt murine thymoma viral oncogene homolog 3 (protein kinase B, gamm	-1.0343102
NM_001017403	LGR6	leucine-rich repeat-containing G protein-coupled receptor 6	-1.0483819
NM_017852	NLRP2	NLR family, pyrin domain containing 2	-1.0551682
NM_000362	TIMP3	TIMP metallopeptidase inhibitor 3	-1.0663665
NM_003613	CILP	cartilage intermediate layer protein, nucleotide pyrophosphohydrolase	-1.0915372
NM_000064	C3	complement component 3	-1.1084812
NM_003013	SFRP2	secreted frizzled-related protein 2	-1.1153132
NM_004625	WNT7A	wingless-type MMTV integration site family, member 7A	-1.1162621
NM_000425	L1CAM	L1 cell adhesion molecule	-1.1770945

NM_021189	CADM3	cell adhesion molecule 3	-1.1790226
NM_004950	EPYC	epiphycan	-1.2010173
NM_001007544	C1orf186	chromosome 1 open reading frame 186	-1.3043064
NM_003914	CCNA1	cyclin A1	-1.3227037
NM_001079858	GPR64	G protein-coupled receptor 64	-1.3843022
NM_005143	HP	haptoglobin	-1.8204692
NM_003890	FCGBP	Fc fragment of IgG binding protein	-1.8866216
NM_006580	CLDN16	claudin 16	-2.3167339
